# ArdA proteins from different mobile genetic elements can bind to the EcoKI Type I DNA methyltransferase of *E. coli K12*^☆^

**DOI:** 10.1016/j.bbapap.2013.12.008

**Published:** 2014-03

**Authors:** Kai Chen, Marcel Reuter, Bansi Sanghvi, Gareth A. Roberts, Laurie P. Cooper, Matthew Tilling, Garry W. Blakely, David T.F. Dryden

**Affiliations:** a EaStCHEM School of Chemistry, The University of Edinburgh, The King's Buildings, Edinburgh EH9 3JJ, UK; b Institute of Cell Biology, School of Biological Sciences, The University of Edinburgh, The King's Buildings, Edinburgh EH9 3JR, UK

**Keywords:** RM, restriction–modification, anti-RM, antirestriction/antimodification, MGE, mobile genetic element, MTase, modification methyltransferase, M subunit, modification subunit, S subunit, sequence specificity subunit, Orf, open reading frame, CD, circular dichroism, GuCl, guanidinium chloride, 2-ME, 2-mercaptoethanol, SEC, size exclusion chromatography, K_d_, dissociation constant, DNA methyltransferase, ArdA protein, DNA mimic, Horizontal gene transfer

## Abstract

Anti-restriction and anti-modification (anti-RM) is the ability to prevent cleavage by DNA restriction–modification (RM) systems of foreign DNA entering a new bacterial host. The evolutionary consequence of anti-RM is the enhanced dissemination of mobile genetic elements. Homologues of ArdA anti-RM proteins are encoded by genes present in many mobile genetic elements such as conjugative plasmids and transposons within bacterial genomes. The ArdA proteins cause anti-RM by mimicking the DNA structure bound by Type I RM enzymes. We have investigated ArdA proteins from the genomes of *Enterococcus faecalis* V583, *Staphylococcus aureus* Mu50 and *Bacteroides fragilis* NCTC 9343, and compared them to the ArdA protein expressed by the conjugative transposon Tn*916*. We find that despite having very different structural stability and secondary structure content, they can all bind to the EcoKI methyltransferase, a core component of the EcoKI Type I RM system. This finding indicates that the less structured ArdA proteins become fully folded upon binding. The ability of ArdA from diverse mobile elements to inhibit Type I RM systems from other bacteria suggests that they are an advantage for transfer not only between closely-related bacteria but also between more distantly related bacterial species.

## Highlights

• Diverse ArdA proteins all target the EcoKI Type I DNA modification enzyme.
• ArdA proteins have variable secondary structure content.
• ArdA all bind equally well to EcoKI despite stability variations.

## 1 Introduction

DNA mimics are a diverse group of proteins expressed by many mobile genetic elements (MGEs) such as bacteriophage, conjugative plasmids encoding multidrug resistance, transposons and even a shrimp virus [1–4]. They can also be found incorporated in bacterial genomes on prophages and other integrative elements. Their function is to bind to DNA-binding sites on target proteins and thereby prevent the target protein from binding to DNA, its normal substrate. Thus these proteins can be considered to be competitive inhibitors.

**Fig. 1:**
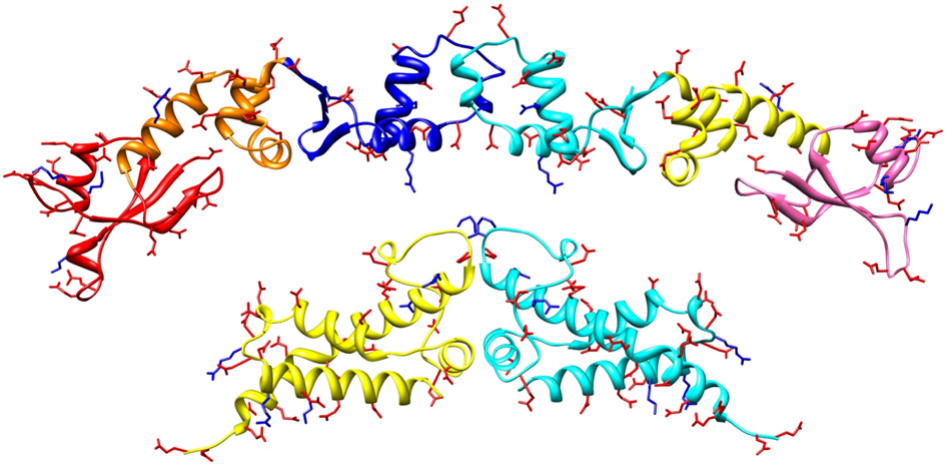
The upper structure shows the dimeric Orf18 ArdA protein with domain 1 coloured red and salmon, domain 2 coloured orange and yellow and domain 3 coloured blue and cyan [9]. The lower structure shows the dimeric structure of the phage T7 Ocr protein (yellow and cyan subunits) [8]. Aspartate and glutamate side chains are shown as red sticks and arginine and lysine side chains as blue sticks. The chord joining the extreme ends of the Orf18 ArdA dimer is ~ 140 Å in length while that joining the ends of the Ocr dimer is ~ 85 Å in length.

The best understood DNA mimics are those which inhibit DNA restriction–modification (RM) enzymes and these “anti-RM” proteins allow the DNA of the MGE to successfully invade the new host bacterial cell [5]. The RM system would usually destroy the invading DNA if it lacks the appropriate pattern of DNA methylation in the DNA sequence recognised by the RM system but the rapid transcription and translation of the DNA mimic overwhelm the RM system [6,7]. Crystal structures of two DNA mimics, the Ocr protein from phage T7 and the ArdA protein from the Tn*916* conjugative transposon, show elongated dimeric proteins whose surfaces are decorated with a great number of aspartate and glutamate side chains in locations corresponding to the phosphate groups on the surface of B-form DNA [8,9], Fig. 1. The mimics fit closely into the DNA binding groove of their targets, the Type I DNA RM enzymes [10,11], and prevent their cleavage of the phage or transposon DNA. Very little sequence variation between Phage T7 Ocr and its few homologues [12] is apparent even though the anti-RM activity is extraordinarily robust to extensive mutagenesis or chemical modification [13–15]. In contrast, putative *ardA* genes are very widespread on MGE within a broad range of bacteria [7,16–19]. ArdA genes are expressed from a novel single-stranded promoter as soon as the conjugative plasmid or transposon enters a new host [7,19]. The predicted ArdA proteins show considerable sequence variation and the genes often encode long N-terminal and/or C-terminal extensions [20]. These differences between Ocr and ArdA may relate to the different life styles of the parent MGE, namely a lytic phage versus a conjugative plasmid or transposon.

It has been shown that ArdA homologues from several MGE infecting *Bacteroides fragilis*, *Enterococcus faecalis* and *Staphylococcus aureus* can operate effectively in vivo against the EcoKI Type I RM system of *Escherichia coli* K12 when expressed in *E. coli* [9]. These ArdA differ considerably in amino acid sequence and essentially can be considered to be rather extreme “variants” of the Tn*916* ArdA whose crystal structure is known [9]. Furthermore their bacterial hosts are classified differently. *E. coli* and *B. fragilis* are Gram-negative members of the Gammaproteobacteria and Proteobacteria Bacteroidetes groups respectively. *E. faecalis* and *S. aureus* are Gram-positive members of the Firmicutes (Lactobacillales and Bacillales groups respectively).

In this paper we explore the interaction in vitro of these ArdA proteins with the core modification methyltransferase (MTase, a complex of two modification, M, subunits and one sequence specificity, S, subunit) of the EcoKI RM system [10,11,21,22] and find that they all bind approximately equally well to the MTase. Binding to a partially assembled but inactive MTase composed of one M and one S subunit [21,22] is also demonstrated. In addition we find that the ArdA proteins display different structural stability ranging from a fully folded form to a partially folded form suggesting that the partially folded ArdA completes its folding upon binding to the MTase. Overall our data suggest that ArdA would assist the spread of MGE between unrelated bacterial species encoding different Type I RM systems.

## 2 Materials and methods

The *ardA* genes selected for overexpression and the molecular biological procedures have been described previously and shown to be active against Type I RM systems in vivo [9,16,20]. The genes selected were open reading frame (Orf) EF2335 from *E. faecalis* V583, Orf SAV0405 from *S. aureus* Mu50 and Orf BF1222 from *B. fragilis* NCTC 9343. The ArdA proteins were overexpressed and purified using DEAE anion exchange chromatography and size exclusion chromatography as described previously for the Orf18 ArdA protein expressed by the conjugative transposon Tn*916* [16]. Extinction coefficients for monomer forms of the ArdA proteins were calculated from their amino acid sequences (Bfr ArdA 39,400 M^− 1^ cm^− 1^; Mu50 ArdA 28,020 M^− 1^ cm^− 1^; V583 ArdA 23,610 M^− 1^ cm^− 1^; Orf18 ArdA 28,020 M^− 1^ cm^− 1^) and used to calculate protein concentration. These coefficients are accurate to +/− 5% [23]. EcoKI MTase was prepared as described previously [21,22]. All experiments were carried out at 25 °C unless otherwise stated. Sequence alignments and determination of amino acid identity and similarity were calculated by PROMALS [24], EMBOSS Needle Alignment (http://www.ebi.ac.uk/Tools/psa/emboss_needle/) and CLUSTAL W2 (http://www.ebi.ac.uk/Tools/msa/clustalw2/). Sequences of Type I RM systems were obtained from REBASE [25].

Circular dichroism (CD) measurements were carried out on a Jasco Model J-180 spectropolarimeter (Jasco Corporation, Tokyo, Japan). All measurements were conducted in 20 mM sodium phosphate, 50 mM NaF, and 0.5 mM 2-mercaptoethanol (2-ME) pH 8.0. NaF has much lower absorption in the far UV than other salts. Far-UV CD spectra were measured in the range of 190–260 nm at protein concentrations of 5.6 to 5.8 μM. All the CD measurements were made at 20 °C using a 1.0 mm pathlength cell and each spectrum was the average of three individual scans run at 20 nm/min, 1 s response time and 1 nm bandwidth. The spectra were corrected for buffer contribution. Secondary structure analysis was performed using CONTIN [26] via the Dichroweb server [27].

Equilibrium unfolding as a function of guanidinium chloride (GuCl) was monitored by tryptophan fluorescence spectroscopy. A stock solution of GuCl was made up in buffer and the precise concentration was determined from the refractive index [28]. Protein (3.5 μM) in 20 mM Tris–HCl 10 mM MgCl_2_ 7 mM 2-ME pH 8.0 was incubated with various concentrations of GuCl at 25 °C and allowed to equilibrate overnight. The fluorescence intensity was then measured for each sample using excitation at 295 nm and emission at 350 nm and 380 nm with 5 nm bandwidths on an Edinburgh Instruments FS900 fluorimeter (Edinburgh Instruments, Livingston, UK). The ratio of intensity at 350 to 380 nm was then fitted to a two-state unfolding model assuming a linear relationship between free energy of unfolding and concentration of GuCl [29]. This ratio compensates for slight differences in the protein concentration between samples.

Size exclusion chromatography (SEC) with a 30 cm long × 0.46 cm diameter Biosep-SEC-S3000 gel filtration column (Phenomenex) was used to characterise the ArdA proteins and their interaction with EcoKI MTase as described previously [30]. The buffer contains 20 mM Tris–HCl, 20 mM MES, 200 mM NaCl, 10 mM MgCl_2_, 0.1 mM EDTA, and 7 mM 2-mercaptoethanol, pH 6.5. The flow rate was set to 0.5 ml/min and the injected sample volume was 40 μl. The column eluate was excited at 295 nm and the fluorescence emission continuously monitored at a wavelength of 350 nm. It is important to note that the pH and sodium chloride concentration used in the chromatography experiments were 6.5 and 0.2 M respectively. The pH is required for the stability of the silica column material. The presence of salt in the buffer is required to prevent non-specific interaction between the protein and the column matrix. Elution profiles were fitted to sums of Gaussian functions using ORIGIN (Originlab Corp., Northampton, MA, USA).

## 3 Results

### 3.1 Sequence comparison

The ArdA proteins from *E. faecalis* V583, *S. aureus* Mu50 and *B. fragilis* NCTC 9343 will be referred to as V583 ArdA, Mu50 ArdA and Bfr ArdA, respectively. The ArdA from Orf18 of Tn*916* will be referred to as Orf18 ArdA. Three of the four proteins examined, Orf18 ArdA, V583 ArdA and Mu50 ArdA, show 74 to 81% sequence similarity and between 56 and 63% identity to each other (Table 1). The Bfr ArdA shows ~ 40% similarity and ~ 25% identity to the other three ArdA sequences and is therefore more distantly related. The variation in sequence is spread throughout the length of the polypeptides but is predominantly located in the second domain (amino acids 62 to 103 of Orf18 ArdA) where identity drops to 50% or even lower (down to 11%) (Supplementary Fig. S1).

### 3.2 Protein purification and secondary structure content of Bfr, V583 and Orf18 ArdA proteins

**Fig. 2:**
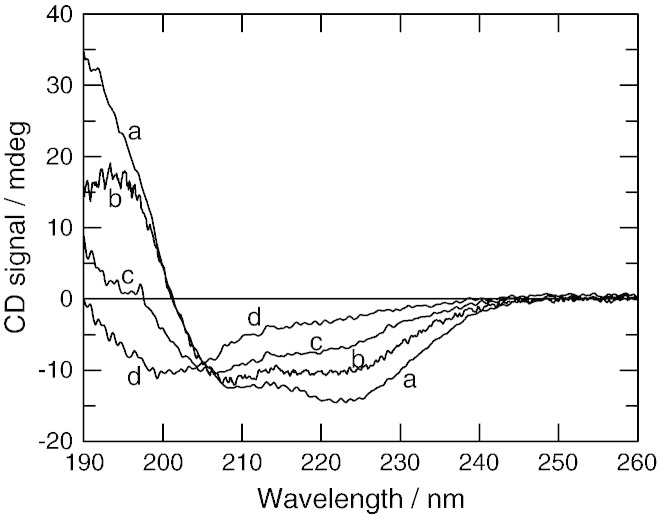
CD spectra of Bfr (a), Orf18 (b), V583 (c) and Mu50 (d) ArdA proteins. The protein concentration was 5.6 to 5.8 μM for each sample.

The Bfr, V583 and Mu50 ArdA proteins could all be purified to homogeneity using the same procedure as used for purifying Orf18 ArdA (size exclusion chromatography profiles for each are described later, SDS-PAGE gels show a single band for each ArdA when stained with Coomassie Blue—data not shown). The crystal structure of Orf18 ArdA shows a mixture of alpha helix (44%), beta sheet (19%) and random coil regions (37%), Table 1. CD spectroscopy was used to assess the secondary structure content of the four ArdA proteins and was found to be surprisingly variable, Fig. 2 and Table 2, given the levels of sequence similarity shown in Table 1. The Orf18 ArdA CD analysis showed the same amount of alpha helix as observed in the crystal structure [9]. Bfr ArdA was almost identical to Orf18 ArdA. V583 ArdA showed more irregular structure than Bfr and Orf18 ArdA proteins with a particularly noticeable drop in alpha helical content. The Mu50 ArdA was the least structured of the proteins showing very low helical content and a large proportion of irregular structure. Domain 2 in the crystal structure is mostly alpha helical or loops; hence the drop in helical content observed with V583 and Mu50 ArdA may reflect the loss of structure of this domain in particular.

### 3.3 Stability of ArdA proteins to denaturation with guanidinium hydrochloride

Fluorescence emission spectra showed a fluorescence maximum due to tryptophan emission at 340 nm for Orf18 ArdA and Bfr ArdA and at 350 nm for V583 ArdA and Mu50 ArdA when excited at 295 nm. These four ArdA proteins have only one conserved tryptophan residue in domain 1 (W23 in Orf18 ArdA and V583 ArdA, W22 in Mu50 ArdA and W28 in Bfr ArdA). This is the only tryptophan in V583 ArdA. An additional tryptophan is found in Orf18 ArdA (W70) and Mu50 ArdA (W91). Both of these tryptophans are in domain 2 but not at conserved positions. Bfr ArdA has four tryptophans in total (the conserved one in domain 1, two unconserved ones in domain 2, W73 and W99, and the fourth in domain 3, W167).

**Fig. 3:**
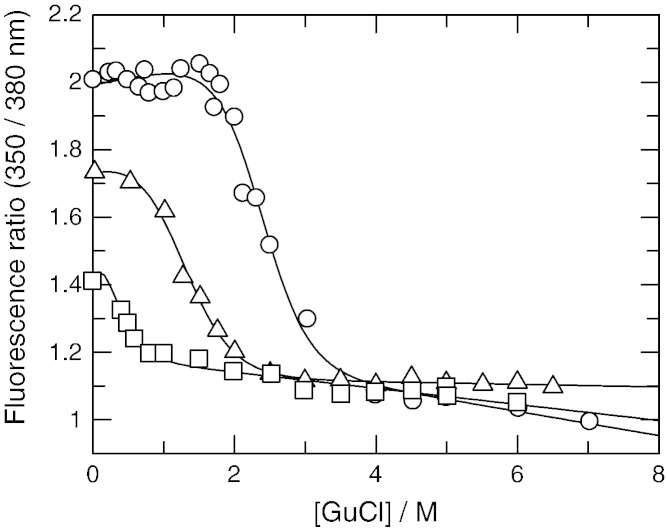
Stability of ArdA proteins in solutions of guanidinium chloride (GuCl). Denaturation curves were determined using the ratio of fluorescence emission intensity at 350 nm to that at 380 nm when excited at 295 nm for Bfr ArdA (circles) V583 ArdA (triangles) and Mu50 ArdA (squares) as a function of GuCl concentration. The fitted lines are from a two-state model of folding [29].

The addition of guanidinium chloride as a denaturant changed the fluorescence intensity and shape of the spectra as the proteins unfolded. As none of the tryptophan residues are located near to the ArdA dimer interface, the change in fluorescence emission observed is due to changes in secondary and tertiary structure only rather than in quaternary structure. Taking the ratio of fluorescence intensity at two emission wavelengths removed minor sample-to-sample variation in protein concentration and allowed calculation of the free energy of unfolding in the buffer in the absence of denaturant, Table 3 and Fig. 3. The folding curves determined by diluting out the denaturant from a sample with fully unfolded protein were identical within experimental error to the unfolding curves (data not shown). This indicates that the transitions are fully reversible. The data were analysed by a two state model. It can be seen that despite the similarity in amino acid sequence between the four proteins their stability varies considerably with Orf18 ArdA being the most stable and Mu50 ArdA the least. All three parameters determined from the unfolding curves; midpoint of the transition, slope of the transition and free energy, showed the same trend as one progressed from the least to the most stable ArdA. There was no evidence for any stable intermediate states in the transition, even with Bfr ArdA protein which contains tryptophan reporter groups in each of the three domains comprising the monomer, such as those observed with multi-domain fragments of Factor H where steps in the transition were obvious [31]. This indicates that the folding/unfolding process is cooperative despite the domain structure. The transition midpoint for Mu50 ArdA was not well defined as there was no plateau region prior to the unfolding transition. The Orf18 ArdA and the Mu50 ArdA proteins are 63% identical and 81% similar in sequence and both have two tryptophan residues, one conserved in domain 1 and the second being unconserved in domain 2, yet they show the most extreme differences in stability. The amino acid sequence between W70 in Orf18 ArdA and W91 in Mu50 ArdA (H90 in Orf18 ArdA) is the most poorly conserved in a pairwise alignment, therefore, if the tryptophan fluorescence from domain 2 in these two proteins is greater than that from domain 1, then the large difference in stability could primarily reflect differences in stability of domain 2 in these two proteins. The difference in stability correlates well with the observed variability in secondary structure content revealed by CD spectroscopy, Fig. 2, and the low levels of sequence identity between the proteins in domain 2 (Supplementary Fig. S1).

### 3.4 Size exclusion chromatography to analyse the stability of the Bfr, V583 and Mu50 ArdA proteins

**Fig. 4:**
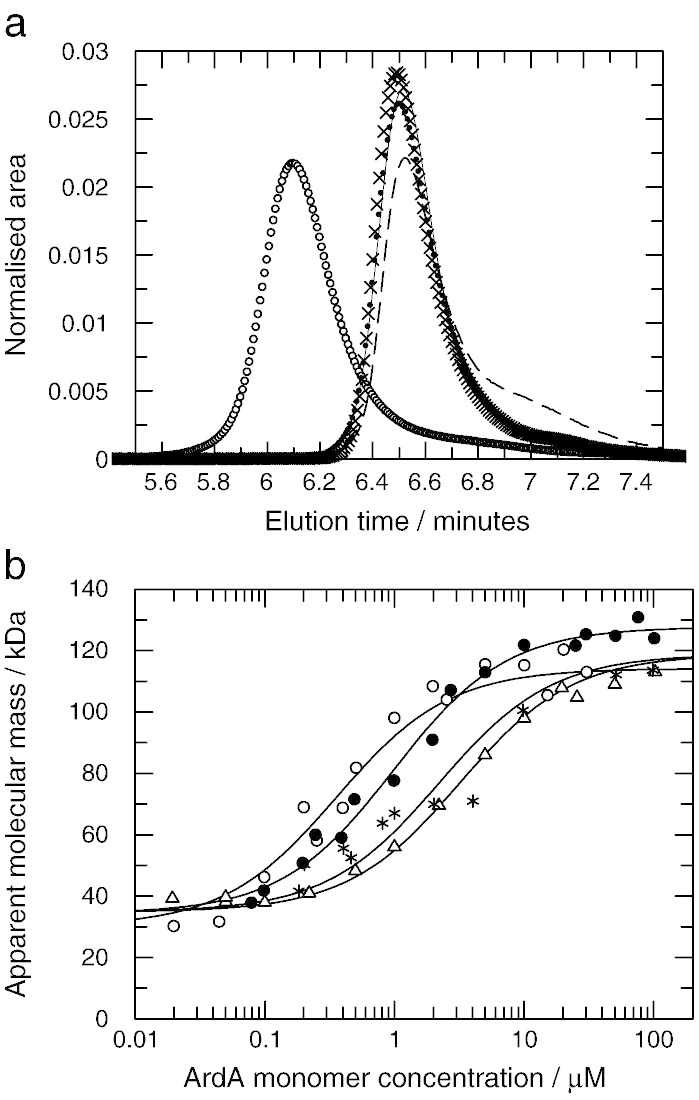
Size exclusion chromatography of ArdA proteins and EcoKI MTase. a. Elution profiles of Bfr ArdA (solid line), Mu50 ArdA (crosses), Orf18 ArdA (dashed line), V583 ArdA (dots) and MTase (open circles) normalised to the same overall area. All proteins were injected onto the column at 2 μM concentration (ArdA monomer concentration or M_2_S_1_ MTase concentration). b. The apparent molecular mass of Bfr ArdA (open circles), Mu50 ArdA (open triangles), Orf18 ArdA (crosses), and V583 ArdA (solid circles) plotted against the monomer concentration. The injected concentrations refer to that of the sample when loaded and ignore dilution by diffusion and mixing during the chromatography. Fitted lines are assuming a monomer–dimer equilibrium and give apparent dissociation constants of 0.36 +/− 0.8, 3.18 +/− 0.14, 2.20 +/− 0.35 and 0.98 +/− 0.13 μM respectively.

Analytical SEC of Bfr, V583 and Mu50 ArdA was performed and compared to the behaviour of Orf18 ArdA, Fig. 4a. Each protein eluted essentially as a single peak at all concentrations investigated except Orf18 ArdA which showed a small amount of a lower molecular mass species (Fig. 4a) but the peaks broadened at lower concentrations due to the presence of a mixture of species differing in quaternary structure (data not shown).

The elution time and apparent molecular mass for each ArdA were determined and plotted against protein concentration injected onto the column, Fig. 4b. The apparent molecular masses at low and high protein concentrations for all three proteins were the same within experimental error and similar to that observed for Orf18 ArdA. Previously, Orf18 ArdA has been found to exist in an equilibrium mixture of monomeric and dimeric forms in solution with a dissociation constant estimated to be ~ 1 μM [16,20]. Both forms are active in antirestriction but the monomeric form has lost the ability to inhibit the MTase modification function of the EcoKI RM system [20]. The Bfr, V583 and Mu50 ArdA proteins appear to behave in the same way as Orf18 ArdA as their molecular mass changes with protein concentration.

An apparent dissociation constant, K_d_, for the dimer was calculated to be 0.36 +/− 0.8, 3.18 +/− 0.14, 2.20 +/− 0.35 and 0.98 +/− 0.13 μM for Bfr, Mu50, Orf18 and V583 ArdA respectively but this value is approximate as firstly, the protein concentration on the column is diluted with respect to the injected concentration and secondly, the dissociation on the column is a non-equilibrium process. The most stable dimer was Bfr ArdA and the least stable were Orf18 ArdA and Mu50 ArdA. The change in apparent K_d_ was less than 10-fold so the difference in free energy between the different proteins is only a few kJ mol^− 1^. The apparent molecular mass of each species determined by SEC, monomer at low concentration and dimer at high concentration, is very different from the masses calculated from the amino acid sequences. This is due to the highly elongated shapes of the monomer and dimer giving larger than expected hydrodynamic radii [16,20,32].

### 3.5 Interaction of ArdA proteins with EcoKI MTase examined via size exclusion chromatography

**Fig. 5:**
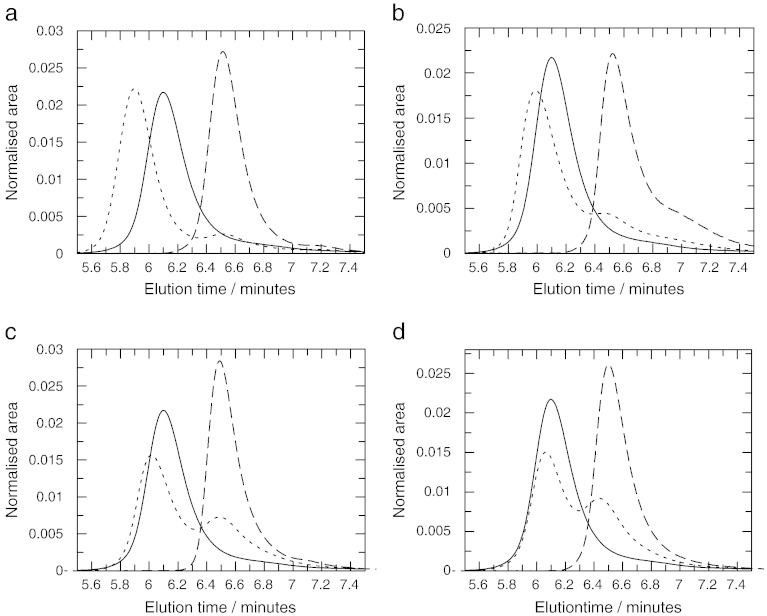
Size-exclusion chromatography analysis of binding between MTase and ArdA to investigate the solution molecular weight. 2 μM concentrations (ArdA monomer or M_2_S_1_ MTase) were injected. The concentrations refer to that of the sample when loaded and ignore dilution by diffusion and mixing during the chromatography. Elution profiles were normalised to the same area. Dotted lines are the mixture of MTase and ArdA; solid lines are MTase; dashed lines are ArdA. a. Bfr ArdA. b. Orf18 ArdA. c. Mu50 ArdA. d. V583 ArdA.

Mixtures of one ArdA dimer (2 μM monomer) with 2 μM MTase were made and subjected to SEC, Fig. 5. All of the mixtures showed the formation of a complex eluting ahead of the MTase elution peak time suggesting binding between the ArdA proteins and the MTase. The mixtures also showed a second peak eluting near the elution time of the free ArdA and this must correspond to unbound ArdA. Fitting two Gaussian curves to these elution profiles allowed a rough estimate of how much ArdA had been bound in each mixture (data not shown). The amount of ArdA bound to the MTase decreased in the order Bfr ArdA > Orf18 ArdA > Mu50 ArdA > V583 ArdA. The elution time of the major peak in the complex of ArdA and MTase could also be used as an indicator of the strength of the ArdA:MTase interaction. This showed the same trend as the Gaussian fitting analysis with the most stable complex eluting at the earliest time. The interaction between ArdA proteins and the MTase is much weaker than the previously characterised interaction between Ocr and the MTase as in such mixtures, no free Ocr was observed; it was all bound by the MTase [30].

### 3.6 Interaction of Bfr and Orf18 ArdA proteins with M_1_S_1_ examined via size exclusion chromatography

**Fig. 6:**
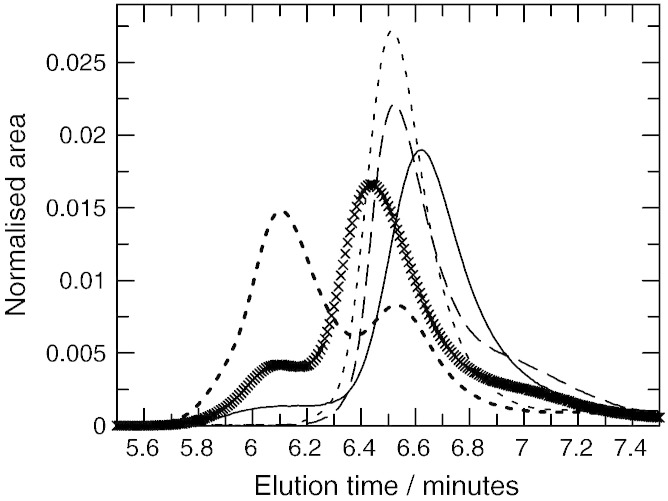
Interaction of ArdA proteins (2 μM monomer injected) with the M_1_S_1_ partially assembled form of the EcoKI MTase (2 μM M_1_S_1_ injected) assessed by size exclusion chromatography. The areas under each curve have been set equal. The concentrations refer to that of the sample when loaded and ignore dilution by diffusion and mixing during the chromatography. M_1_S_1_ (solid line), Bfr ArdA (dotted line), Orf18 ArdA (dashed line), M_1_S_1_ mixed with Bfr ArdA (bold dotted line) and M_1_S_1_ mixed with Orf18 ArdA (crosses).

The MTase has previously been shown to dissociate at low concentrations as it passes through a size exclusion column [21,22,30]. It dissociates into an M_1_S_1_ form and an M subunit with a dissociation constant of between 15 and 90 nM. The behaviour of mixtures of the M_1_S_1_ form and two of the ArdA proteins on SEC was investigated. Mixtures of 2 μM M_1_S_1_ with 2 μM monomer of Bfr ArdA or Orf18 ArdA were made and subjected to SEC, Fig. 6. The Bfr ArdA bound well to M_1_S_1_ (main peak at 6.1 min with a smaller peak at 6.5 min due to uncomplexed material) whereas the Orf18 ArdA bound less well with the bulk of the material eluting at 6.4 min ahead of the individual ArdA and M_1_S_1_ peaks. A small amount of material eluted at 6.1 min. The elution of material ahead of the individual ArdA and M_1_S_1_ peaks indicated that M_1_S_1_ can bind ArdA but the affinity for M_1_S_1_ was higher for Bfr ArdA than for Orf18 ArdA. We assume that Mu50 and V583 ArdA can also interact in a similar manner with M_1_S_1_.

## 4 Discussion

The size exclusion data clearly show that the ArdA proteins exist in two forms in agreement with previous data on Orf18 ArdA [16,20]. Ultracentrifugation has previously defined these as monomer and dimer forms of ArdA [20]. Furthermore, the size exclusion data show that ArdA proteins can interact with the core MTase of EcoKI and the M_1_S_1_ partially assembled core. Preliminary data obtained using both isothermal titration calorimetry and quartz crystal microbalance support the existence of these interactions under a range of pH and ionic strength conditions (G.A. Roberts and M. Reuter, unpublished data). All of the data lead to the conclusion that a complex set of multiple equilibria between the different quaternary structure forms of EcoKI and of ArdA will exist. The determination of the dissociation constants between all of these different species will be impossible with the current methods and would require a technique such as mass spectrometry to quantify every species present in a mixture [33]. However, from the presented data, we conclude that the strength of the binding between ArdA and the MTase decreases in the order Bfr ArdA > Orf18 ArdA > V583 ArdA ~ Mu50 ArdA. This correlates with the stability and secondary structure content of the proteins (Bfr ArdA ~ Orf18 ArdA > V583 ArdA > Mu50 ArdA).

The affinity of ArdA for EcoKI MTase is poorer than that of the DNA target sequence for the MTase where affinities of the order of 1 to 10 nM were observed [34,35]. Nor do the ArdA proteins bind as tightly to the EcoKI MTase as the Ocr antirestriction protein [30]. EcoKI MTase binds very tightly (K_d_ of ~ 50 pM) to Ocr and the interaction is only weakened when Ocr is extensively neutralised by chemical modification [15] or large-scale mutagenesis [14]. Single or double mutations of Ocr had no effect on the interaction between Ocr and EcoKI [13]. Assuming that no additional protein component of ArdA is missing in our protein preparations, an assumption which is reasonable given that the *ardA* genes are not part of any operon and are transcribed from a single promoter [7,19], then the ArdA proteins are not such good mimics of the DNA bound by the MTase as the Ocr protein. This is in agreement with data on ArdA from the conjugative plasmid ColIb-P9 [17,18]. Our conclusion is perhaps unexpected as ArdA has approximately the same number of negative charges per unit length as Ocr. Orf18 ArdA, for example, has 14 aspartate residues and 24 glutamate residues but only 3 arginine and 3 lysine residues per monomer and each monomer is ~ 7 nm in length. Ocr has 17 aspartate and 17 glutamate residues but only 4 arginine and 2 lysine residues per monomer and each monomer is ~ 4 nm in length so one might expect ArdA to be as effective as Ocr if only electrostatic interactions where involved in the DNA mimicry. Comparison of the shape of Ocr [8] and ArdA [9] implies that Ocr is a better fit into the MTase than the ArdA proteins. Thus shape complementarity in addition to electrostatic attraction is required for very tight binding as previously concluded from a study of chemical modification of Ocr [15].

The observation that the secondary structure and stability of the ArdA proteins varied greatly was unexpected given the considerable levels of sequence similarity particularly between the Mu50, V583 and Orf18 ArdA proteins. The level of similarity is lowest in the predominantly alpha helical Domain 2 which would correlate with the low level of alpha helical structure observed in the least stable Mu50 ArdA. These data may indicate a varying amount of flexibility in the different proteins with Mu50 ArdA being the most flexible DNA mimic. Given that all of the proteins bind similarly to the EcoKI MTase, we speculate that the binding event induces unstructured or weakly structured parts of ArdA to fold. These differences in structure and dynamics could be investigated further by, for example, ion-mobility mass spectrometry [36].

The amino acid sequences of the subunits of Type I RM enzymes usually differ considerably even when isolated from the same bacterial species. The levels of identity are around ~ 30% at best and confined to conserved motifs required for substrate and cofactor binding and catalysis [5,37,38]. The only exceptions to this are when the enzymes belong to the same “family” and exhibit subunit complementation, DNA hybridisation and antibody cross reactivity. In a family, sequence identity is nearly 100% for the M subunits and the R subunits. The Type I RM systems encoded by the bacterial species used as the sources of the ArdA proteins examined here, namely, *E. faecalis*, *B. fragilis* and *S. aureus*, do not belong to the same family when assessed by sequence conservation (data not shown, sequences of Type I RM systems obtained from REBASE [25]). Given that the ArdA proteins examined here are from unrelated species but all work against the EcoKI system of *E. coli* K12 [10,11], then it would seem to be a reasonable prediction that they will also work against the Type I RM systems from *E. faecalis*, *B. fragilis* and *S. aureus* and, indeed, against any Type I RM system. Thus the source of the ArdA does not seem to be important for their function as anti-RM proteins. This retention of function could aid horizontal gene transfer of mobile genetic elements containing *ardA* genes across species boundaries [39].

## Figures and Tables

**Table 1:** Percentage similarity (above diagonal) and identity (below diagonal) for the four ArdA proteins.

	Orf18 ArdA	V583 ArdA	Mu50 ArdA	Bfr ArdA
Orf18	100	74	81	40
V583	56	100	78	43
Mu50	63	62	100	43
Bfr	24	30	26	100

**Table 2:** Secondary structure content of the ArdA proteins determined by analysis of the CD spectra using CONTIN [26,27]. Secondary structure content of the crystallised Orf18 ArdA is from [9].

Protein	Helix %	Sheet %	Turns and irregular %
Orf18 ArdA crystal	44	19	37
Orf18 ArdA	39	12	49
V583 ArdA	17	29	54
Mu50 ArdA	9	33	58
Bfr ArdA	42	11	47

**Table 3:** Analysis of unfolding as a function of guanidinium chloride concentration using a two-state model. The free energy of unfolding in the absence of denaturant is calculated by multiplying the slope of the transition by the midpoint of the transition. The temperature was 298 K.

Protein	Midpoint of transition/M	Slope of transition/RT	Free energy of unfolding in buffer/kJ mol^− 1^
Orf18 ArdA^a^	2.88 +/− 0.06	5.24 +/− 1.37	37.2
V583 ArdA	1.22 +/− 0.16	2.92 +/− 0.46	8.8
Mu50 ArdA	0.30 +/− 0.75	2.31 +/− 0.85	1.7
Bfr ArdA	2.32 +/− 0.08	3.36 +/− 0.65	19.3

a Analysis from [16].
