# Supplementary material for: ArdA proteins from different mobile genetic elements can bind to the EcoKI Type I DNA methyltransferase of E. coli K12
Source: Biochim Biophys Acta. 2014 Mar;1844(3):505–11. doi: 10.1016/j.bbapap.2013.12.008 (PMC3969726; doi:10.1016/j.bbapap.2013.12.008)
Supplement: Supplementary file 1 — Supplementary figure and table. [file mmc1.doc]

**Supplementary Information**

**ArdA proteins from different mobile genetic elements can bind to the EcoKI Type I DNA methyltransferase of *E. coli K12*.**

Kai Chen, Marcel Reuter, Bansi Sanghvi, Gareth A. Roberts, Laurie P. Cooper, Matthew Tilling, Garry W. Blakely and David T.F. Dryden

**Supplementary Figure S1. PROMALS [22] secondary structure (ss) and amino acid sequence alignment (aa) for the investigated ArdA proteins.** The three domains of Orf18 ArdA are defined by aa blocks 1-61, 62-103 and 104-165 and a blank space is introduced into the protein sequences at the boundaries between the domains.

Mu50 ArdA 1 -----M-EMKVYVANLGRYNEGELVGAWFTPPI--DEEEMAERIG---LNEDYEEYAIHDFEL--PFDVD 57

Orf18 ArdA 1 -----MDDMQVYIANLGKYNEGELVGAWFTFPI--DFEEVKEKIG---LNDEYEEYAIHDYEL--PFTVD 58

V583 ArdA 1 -----MEQMRVYIANLGKYNEGELVGAWFTPPV--DFDEVKERIG---LNDDYEEYAIHDYEL--PFEID 58

Bfr ArdA 1 MEAVTLSEARVYVGTYNKYNNGSLFGKWLDLSDYSDKDEFMEACRELHKDDQDPEFMFQDYENIPEALIS 70

Consensus aa: .....h.-hpVYltshsKYNpGpLhG.Whs.s...Db-EhbE.h....bsDp..E@hhpDYE....h.ls

Consensus ss: hhhhhhhhhhhh eeeeeeee hhhhhhhhhhh eeee

Mu50 ArdA 58 EYT PISEINRLCEAIQEIEGTPIYNELKEIQGMWFSSLEELLE-----NKED IHCYSDCDSMEDVARYYV 122

Orf18 ArdA 59 EYT SIGELNRLWEMVSELP-EELQSELSALLTH-FSSIEELSE-----HQED IIIHSDCDDMYDVARYYI 121

V583 ArdA 59 EYT SIEEINRLCGLAEELEGTPIGEVASEIQHAFFNSFEEMVE-----HVDD IVYYPDCNDMEDLAYQMV 123

Bfr ArdA 71 ESW LSEKFFELRDAIEKLSETQ--QEAFFVWCDHHNSDISEEDADDLISSFE DEYQGEYKDEEDYAYEIV 138

Consensus aa: E.h ...ch.cL.-hlpcL..p.l.pEh..lbh..@sS.bpb.-.....ppb- ..h.t-hcDbbDhA.bhl

Consensus ss: hhhhhhhhhhhhh hhhhhhhhhhhhh hhhhhh hhhhhhhh hhh hhhhhhhhh

Mu50 ArdA 123 EETGQLGEVPSNLQNYIDYQSLGRDMEIEGNYLVTSHGVFEYCQ 166

Orf18 ArdA 122 EETGALGEVPASLQNYIDYQAYGRDLDLSGTFISTNHGIFEIVY 165

V583 ArdA 124 NE-GYLGDAPENFVRYFNYSSFARDLEIEGNYLVTNRGIFEYPI 166

Bfr ArdA 139 EQC---YDLPEFAKTYFDYSAFARDLFITDYWMD-NGFVF-RCA 177

Consensus aa: EphG.L.-lP..hpsYhDYpt@tRDL.lps.@hsTN..lFE.hh

Consensus ss: hhh hh hhhhhhhhhhhh hhh eeeee eeeeee

Consensus amino acid symbols are: conserved amino acids are in bold and uppercase letters; aliphatic (I, V, L): l; aromatic (Y, H, W, F): @; hydrophobic (W, F, Y, M, L, I, V, A, C, T, H): h; alcohol (S, T): o; polar residues (D, E, H, K, N, Q, R, S, T): p; tiny (A, G, C, S): t; small (A, G, C, S, V, N, D, T, P): s; bulky residues (E, F, I, K, L, M, Q, R, W, Y): b; positively charged (K, R, H): +; negatively charged (D, E): -; charged (D, E, K, R, H): c.

**Amino acid identity between each domain of the ArdA proteins.**

Domain 1

Orf18 100.00 83.61 76.67 29.51

V583 83.61 100.00 76.67 31.15

Mu50 76.67 76.67 100.00 26.67

Bfr 29.51 31.15 26.67 100.00

Domain 2

Mu50 100.00 50.00 42.86 14.29

V583 50.00 100.00 38.10 11.90

Orf18 42.86 38.10 100.00 21.95

Bfr 14.29 11.90 21.95 100.00

Domain 3

Mu50 100.00 61.29 52.46 26.32

Orf18 61.29 100.00 39.34 28.07

V583 52.46 39.34 100.00 42.11

Bfr 26.32 28.07 42.11 100.00
